# Distribution of energy and macronutrient intakes across eating occasions in European children from 3 to 8 years of age: The EU Childhood Obesity Project Study

**DOI:** 10.1007/s00394-022-02944-6

**Published:** 2022-08-05

**Authors:** Vanessa Jaeger, Berthold Koletzko, Veronica Luque, Natàlia Ferré, Dariusz Gruszfeld, Kinga Gradowska, Elvira Verduci, Gian Vincenzo Zuccotti, Annick Xhonneux, Pascale Poncelet, Veit Grote

**Affiliations:** 1 Division of Metabolic and Nutritional Medicine, Department of Paediatrics, Dr. von Hauner Children’s Hospital, LMU University Hospitals, LMU - Ludwig-Maximilians-Universität Munich, Lindwurmstr. 4, 80337 Munich, Germany; 2 Paediatrics Research Unit, Universitat Rovira I Virgili, IISPV, Reus, Spain; 3 Serra Hunter Fellow, Universitat Rovira I Virgili, IISPV, Reus, Spain; 4 Neonatal Department and Neonatal Intensive Care Unit, Children’s Memorial Health Institute, Warsaw, Poland; 5 Department of Paediatrics, Vittore Buzzi Children’s Hospital, University of Milan, Milan, Italy; 6 Centre Hospitalier Chretien St. Vincent, Rocourt, Liège‑Rocourt, Belgium; 7 Department of Paediatrics, University Children’s Hospital, Queen Fabiola, Université Libre de Bruxelles, Brussels, Belgium

**Keywords:** Children, Chrono-nutrition, Time-varying, Energy, Macronutrients, Dietary pattern

## Abstract

**Purpose:**

We aimed to characterize the distribution of energy and macronutrient intakes across eating occasions (EO) in European children from preschool to school age.

**Methods:**

Data from 3-day weighed food records were collected from children at ages 3, 4, 5, 6 and 8 years from Belgium, Germany, Italy, Poland and Spain. Food intakes were assigned to EO based on country-specific daytimes for breakfast, lunch, supper and snacks (morning, afternoon). The average energy and nutrient intakes were expressed as percentage of total energy intake (%E). Nutrients were additionally expressed as percentage per EO (%E_EO_). Foods were assigned to food groups; variation in intake was calculated via coefficient of variation (CV). We analyzed age trends in diurnal intake using mixed-effects beta regression.

**Results:**

The 740 healthy children included in the analysis consumed the largest proportion of daily energy at lunch (31%E ± 8, M ± SD) and supper (26%E ± 8), followed by breakfast (19%E ± 7) and snacks [afternoon (16%E ± 8); morning (8%E ± 7)], with the most variable intake at morning snack (CV = 0.9). The nutrient composition at lunch and supper was highest for fat (36 ± 9%E_Lunch_; 39 ± 11%E_Supper_) and protein (18 ± 5%E_Lunch_; 18 ± 6%E_Supper_) and at breakfast and snacks for carbohydrates (54 ± 12%E_Breakfast_; 62 ± 12%E_Snacks_). High-sugar content foods were consumed in relatively large proportions at breakfast and snacks. Food intakes varied significantly with age, with lower snack intakes at later ages (*p* < 0.001).

**Conclusion:**

Possibly unhealthy EOs with high-fat intakes and high-sugar-content foods were observed. Changes in nutrient composition of EOs may be beneficial for health.

**Trial registry:** ClinicalTrials.gov: NCT00338689; 19/June/2006.

**Supplementary Information:**

The online version contains supplementary material available at 10.1007/s00394-022-02944-6.

## Introduction

Chrono-nutrition is an emerging field in nutrition science which does not only study what is eaten but also takes the timing, regularity and frequency of eating into account [1]. Research in children and adolescents has focused on breakfast skipping [2, 3] or late night eating [4, 5] and its possible health effects.

The diet of young children is markedly influenced by the family’s eating behaviour [6]. Eating behaviour develops already during the first and second year of life, particularly after introduction of complementary feeding, and is likely to track into mid-childhood and older ages [7]. Infants and young children typically eat more frequently than older children and adults [8]. When children get older, eating occasions (EO) in western countries are usually three main meals per day (breakfast, lunch and supper) and an additional one-to-three snacks between main meals. The quantity and composition of food as well as the time when food is consumed varies markedly across countries [9–11]. Furthermore, first results of studies examining time-varying food intake suggest that dietary intakes at different times of the day might have differential effects on weight gain [12, 13].

Studies investigating eating behaviour in children have focused mainly on one [3, 14] or few EOs [15]. In particular, breakfast consumption and its health consequences have been studied [16, 17]. Food and nutrient intake at one meal is affected by the meal and snacks eaten before. Also, how food intake is distributed within a day may vary with age. Better understanding of eating habits across the day might contribute to strategies for improving dietary habits and health in children. Therefore, we aimed to describe the energy and macronutrient (total fat, carbohydrate and protein) intake at different EOs over the day in children aged 3 to 8 years in five European countries. Differences between countries, sex and age groups and skipping of EOs are analyzed.

## Materials and methods

### Study design and population

This study used dietary data collected as part of the European Childhood Obesity Project (CHOP) randomized intervention trial. The intervention examined the effect of different protein content in infant formula on growth and later obesity risk [18, 19]; apart from the intervention groups, one-third of all included children were fully breastfed for at least 3 months of life. Between 2002 and 2004, 1678 infants in Belgium (Liège and Brussels), Germany (Munich and Nuremburg), Italy (Milano), Poland (Warsaw) and Spain (Tarragona and Reus) were enrolled during their first 8 weeks of life (median 2 weeks) and followed up until 11 years of age. Only healthy, full-term and singleton infants were included in the trial. The trial was conducted in accordance with the Declaration of Helsinki, approved by the ethical committee from each participating site, and registered (NCT00338689, clinicaltrials.gov). Parents provided written informed consent before enrolment. At the age of 8 years, children also provided assent to further follow-up.

### Dietary assessment

Dietary data with predefined EOs were collected at the ages 3, 4, 5, 6 and 8 years using weighed and estimated dietary records for three consecutive days (2 weekdays and 1 weekend day) completed by parents or caretakers. Food and leftovers were weighed using food scales (Unica 66006; Soehnle, Murrhardt, Germany). In case no weighing was possible, an atlas of food pictures was used to support the estimation of portion sizes.

Dietary records were entered in a software specifically developed and designed for this study. Nutritional products and their content were primarily based on the German food composition database BLS 2.3 (Bundeslebensmittelschlüssel) and were enriched by information from product labels, manufacturers or national food databases of participating countries, if necessary. For analysis, nutritional values of products were updated to BLS 3.01. Quality checks of collected dietary records were executed by trained dieticians on several stages following standard operating procedures [20, 21]; all records were checked by a dietician together with the parents at each study visit. Foods and beverages reported by parents were assigned to food groups and summarized to main groups. A list with the types of foods in each food group are detailed in the Supplementary Table S1.

Misreporting of energy intake was considered based on the ratio of reported energy intake and estimated energy requirements as described in detail elsewhere [22]. Misreports were identified but not excluded as recommended [23].

### Eating occasions (EO)

An EO is any event when foods or beverages are consumed. The term EO is a neutral definition to examine eating patterns including meals and snacks. In this analysis, EOs were predefined (before data collection) according to typical time slots and typical foods in each country in 3 meals (breakfast, lunch and supper) and 3 snacks (morning, afternoon and evening). All food intakes were then assigned to one of the six EOs above. Thus, also country-specific distinctions of times of EOs on weekends and weekdays were taken into account.

Skipping of EOs was defined as subjects who have not eaten an EO throughout all days of a food record. Intake from subjects skipping an EO was included in the analysis with zero calories.

### Covariates

At each time point, weight and height were measured by trained study personal. Sex, smoking during pregnancy and parental education were collected by questionnaire at baseline. The latter was classified according to the International Standard Classification of Education [24] into low, medium and high level of education. Maternal body mass index [BMI; weight (kg)/height (m^2^)] was calculated from self-reported pre-pregnancy weight and from height measured at baseline. BMI was classified in weight groups according to the World Health Organisation [25].

### Statistical analysis

Subjects with dietary data on at least one out of five time points (3, 4, 5, 6 and 8 years) were included in the analysis as described previously [18]. The average intake (kcal/day) was calculated for energy and energy from macronutrients (carbohydrate, protein and fat) from the dietary records for each time point. Each nutrient was expressed either as percentage contribution towards total daily energy intake (TEI;%E) or as its percentage contribution towards total energy intake for each EO (%E_EO_). The nutrient composition in%E_EO_ facilitated the assessment of nutrient composition at each EO independent of the amount of energy each EO is contributing towards TEI. The average intakes for energy and energy from nutrients were summarized by age and country unless otherwise indicated.

Data are presented as arithmetic mean (standard deviation) for continuous variables and as counts (%) for categorical variables. CV (standard deviation/mean) was used to assess variability independent of size of meal consumed.

Age effects for energy and nutrient intakes were estimated using mixed-effects beta regression which is suitable to model continuous proportional outcomes with repeated measurements. Intake from subjects skipping an EO was transformed according to (*Y* × (*n*-1) + 0.5)/*n*, where *n* is the sample size and *Y* energy intake, as recommended [26]. Age effects for afternoon and morning snacks were combined to “snacks” due to a higher number of subjects skipping an EO. Mixed-effects models with a logit link were used with child-specific random intercept and slope over age. Age was added as fixed effects either as continuous variable or in case of non-linearity as piecewise linear splines (energy and fat: splines for lunch with knots at 5 and 6 years; carbohydrate: splines for lunch with knot at 5 years; protein: splines for lunch with knot at 4 year and snacks with knot at 5 years). Energy and energy from nutrients for each EO (% and %E, respectively) were used as outcomes. The models were adjusted for misreporting, TEI, country and the interaction of TEI and country. The interaction was included as the increase in TEI over age differed by country. For macronutrients TEI was replaced by energy from total carbohydrate (kcal), total protein (kcal) and total fat (kcal), respectively. Model specifications were checked graphically by depiction of residuals. Sensitivity analyses were performed by exclusion of 2.5% of highest and lowest intake for each EO and energy or energy from nutrients. Regression results were plotted using predicted values. Sex differences in energy and nutrient intakes (in %E) at each EO were tested by t-test with food intakes averaged over age for each subject to avoid dependent observations. All statistical analysis were performed by R studio version 4.0.4 [27], and the package “glmmTMB”, “DHARMa” and “ggeffects” were applied. Statistical testing was defined as significant for *p *< 0.05 with Bonferroni adjustment.

## Results

We included 740 healthy subjects (53% girls) with 2563 food records in this analysis. Subjects participated on average in 3.5 out of 5 time points, whereas 33% of subjects participated in all 5 time points. 97% of all food protocols were recorded for 3 days as intended. Most of the subjects were recruited in Spain (28%), followed by Italy (27%), Poland (17%), Germany (14%) and Belgium (13%). The average participation rate was highest in Italy (4 out of 5 visits) and lowest in Poland (2 out of 5 time points). A third of the children belonged to the initial observational breastfed group and 68% to the intervention group. Half of subject parents had a medium education level and participation rate at all 5 time points was higher if parents had a medium or high education level. Maternal pre-pregnancy BMI was classified as normal-weight in 63% of the mothers, as overweight in 20% and obese in 8% of all mothers. One-third of the mothers smoked during pregnancy. Twenty-four percent of the subjects were at risk of overweight or obesity with a BMI z-score above 1, with highest rate at 8 years of age (30% of subjects). Average EO frequency decreased from 3 to 8 years from 5.7 EOs to 5.1 EOs per day (excluding EOs consisting of water or unsweetened tea).

The mean dietary intake for the study population stratified by age group and the total population is described in Table 1. The average daily energy consumption increased from 1160 kcal at 3 years to 1547 kcal at 8 years, while the energy intake per kg body weight decreased considerably, but the contribution of macronutrients to energy were very similar for each age group. The highest average total energy was consumed in Poland (1449 ± 239 kcal) followed by Spain (1403 ± 293 kcal), Italy (1305 ± 273 kcal), Belgium (1253 ± 250 kcal) and Germany (1237 ± 297 kcal). The total amount of energy from carbohydrate was highest in Germany (756 ± 352 kcal; 61%E) and for protein (246 ± 59 kcal; 18%E) and fat (559 ± 149 kcal; 40%E) in Spain (Supplementary Table 2 and 3). Dietary data were identified as under-reported in 13% and as over-reported in 12% of all observations. The number of plausible reports was highest in Germany with 81% of dietary records. Highest percentages of over-reporting and under-reporting were seen in Poland (16% and 11% of dietary records, respectively) and Spain (14% and 13% of dietary records, respectively).

**Table 1:** Mean energy and macronutrient intake of children aged 3 to 8 years

Age in years	3 (*n* = 570)	4 (*n* = 533)	5 (*n* = 489)	6 (*n* = 525)	8 (*n* = 446)	Total (*N* = 740)^a^
Energy						
Kcal/day	1160 ± 240	1268 ± 235	1343 ± 249	1432 ± 249	1547 ± 288	1340 ± 284
Kcal/kg^b^	80 ± 18	76 ± 16	71 ± 15	66 ± 14	56 ± 13	70 ± 17
Carbohydrates						
g/day	150 ± 46	160 ± 37	172 ± 49	183 ± 43	192 ± 49	170 ± 47
%E^c^	52 ± 16	51 ± 10	51 ± 13	51 ± 10	50 ± 11	51 ± 12
Protein						
g/day	45 ± 12	48 ± 12	50 ± 13	54 ± 13	59 ± 15	51 ± 14
%E^c^	16 ± 3	15 ± 3	15 ± 3	15 ± 3	15 ± 3	15 ± 3
Fat						
g/day	45 ± 12	50 ± 13	53 ± 13	56 ± 14	62 ± 17	53 ± 15
%E^c^	35 ± 6	35 ± 6	35 ± 6	35 ± 6	36 ± 6	35 ± 6

Values are presented as mean ± standard deviation.

^a^740 subjects with 2563 observations in total

^b^48 subjects had no weight measurements

^c^Percentage values are based on total energy intake per day

The relative contribution of energy and energy from macronutrients at each eating occasion is summarized in Table 2. Most energy was consumed at lunch, followed by supper and breakfast. The snack during afternoon provided more energy than the morning snack. Snack intakes contributed 24% of TEI and, hence, a greater contribution than breakfast (19%). The highest variation in mean intake (CV) was seen at morning snacks, especially for protein and fat.

**Table 2:** Energy and macronutrient intake by eating occasion in percentage of daily total energy intake and of total energy intake from macronutrient with coefficient of variation (CV) in 740 children with food protocols (*N* = 2563) at 3, 4, 5, 6, and 8 years of age and from 5 countries (Belgium, Germany, Italy, Poland, and Spain)

	Energy		Carbohydrates		Protein		Fat	
	Mean ± SD (%)	CV	Mean ± SD (%E)	CV	Mean ± SD (%E)	CV	Mean ± SD (%E)	CV
Breakfast	18.9 ± 6.8	0.4	20.3 ± 8.0	0.4	17.7 ± 7.5	0.4	17.9 ± 8.7	0.5
Morning snack	7.9 ± 7.4	0.9	9.9 ± 8.8	0.9	5.3 ± 6.1	1.1	6.3 ± 7.6	1.2
Lunch	30.9 ± 8.4	0.3	28.0 ± 9.2	0.3	36.5 ± 10.3	0.3	32.4 ± 11.1	0.3
Afternoon snack	16.4 ± 7.6	0.5	19.9 ± 8.9	0.4	10.4 ± 6.4	0.6	14.3 ± 9.1	0.6
Supper	25.8 ± 8.1	0.3	22.0 ± 8.9	0.4	30.0 ± 10.5	0.4	29.0 ± 11.1	0.4

*CV* coefficient of variation, *SD* standard deviation

**Fig. 1:**
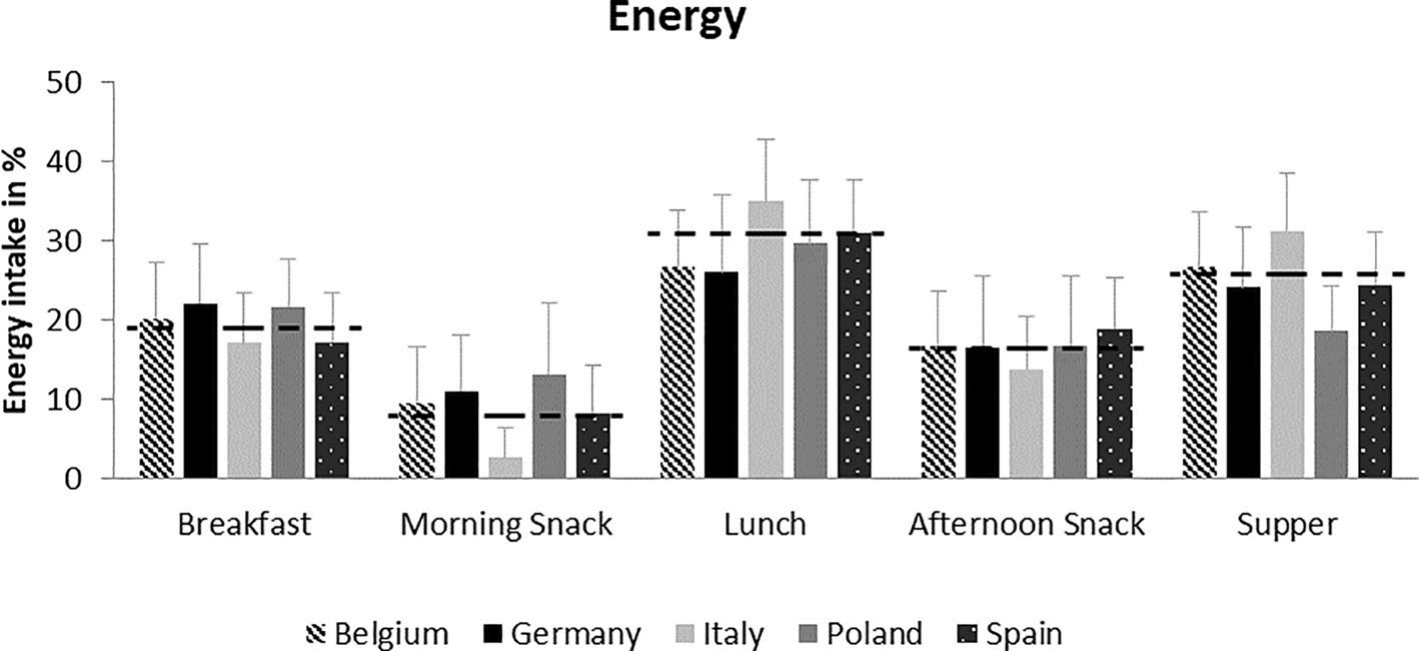
Average energy intake in percentage of total intake per eating occasion in children followed at 3, 4, 5, 6, and 8 years of age in Belgium (*n* = 97), Germany (*n* = 106), Italy (*n* = 201), Poland (*n* = 126), and Spain (*n* = 210). The dashed lines indicate the mean of each eating occasion across all countries (*N* = 740)

In Fig. 1 the average intake of energy is displayed for each country. Breakfast (22 ± 6%) was larger than supper (19 ± 6%) in Poland, whereas in Belgium, lunch and supper were consumed with similar size. Morning snack in Italy was very small (3 ± 4%), but lunch (35 ± 8%) and supper (26 ± 7%) were higher than in the other countries.

**Fig. 2:**
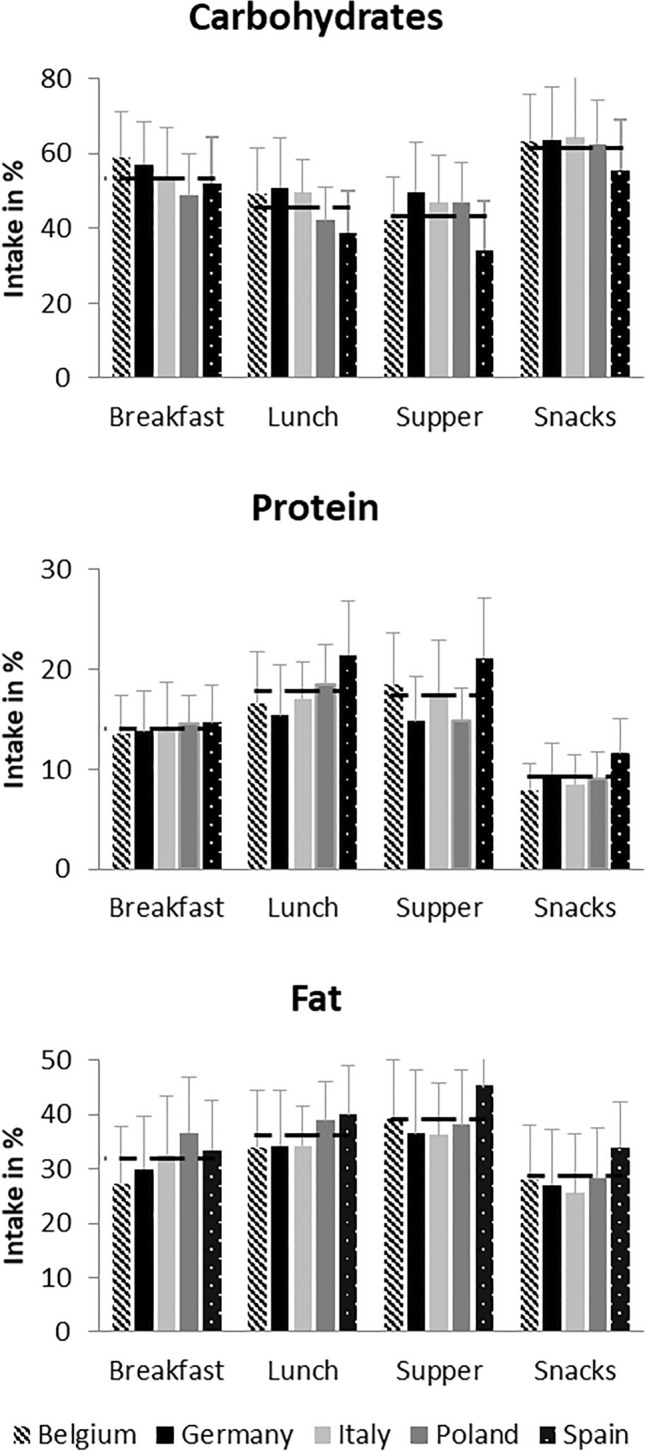
Average macronutrient intake as percentage of total energy intake per eating occasion (%EO) of children followed at 3, 4, 5, 6, and 8 years of age in Belgium (*n* = 97), Germany (*n* = 106), Italy (*n* = 201), Poland (*n* = 126), and Spain (*n* = 210). The dashed lines indicate the mean of each eating occasion across all countries

Overall and country-specific average nutrient distribution for each EO over age is depicted in Fig. 2. Lunch and supper were characterized by high intakes of fat (lunch: 36.3 ± 9.1%E_Lunch_; supper:39.1 ± 10.9%E_Supper_) and protein (lunch: 18.1 ± 5.2%E_Lunch_; supper: 17.7 ± 5.6%E_Supper_), whereas breakfast and snacks were characterized by high proportions of carbohydrates (breakfast: 53.7 ± 11.7%E_Breakfast_; snacks: 61.9 ± 12.2%E_Snacks_). In Spain, carbohydrate intakes were less than in the other countries, especially at lunch (39%E_Lunch_), supper (34%E_Supper_) and in snacks (55%E_Snacks_). In contrast, in Spain, more protein-containing foods were consumed at lunch (21%E_Lunch_), supper (21%E_Supper_) and snacks (12%E_Snacks_) than in most other countries. Greater amounts of fat-containing foods were more often consumed in Spain at all EOs and in Poland at breakfast (37%E_Breakfast_) and lunch (39%E_Lunch_) than in the remaining countries.

The most frequently consumed food groups per EO are presented in Table 3. Breakfast was characterized by high-sugar-content products, milk products and cereals; snacks had similar characteristics, but fruits and beverages were often additionally consumed. Vegetables, cereals, fats and meat were typically eaten at lunch and supper.

**Table 3:** Frequency of the five most often consumed food groups as percentage of total food groups at each eating occasion

Breakfast		Morning snack		Lunch		Afternoon snack		Supper	
Food group	%	Food group	%	Food group	%	Food group	%	Food group	%
High-sugar-content products	31	High-sugar-content products	22	Vegetables & vegetable products	17	High-sugar-content products	30	Cereals	16
Milk & milk products	30	Cereals	16	Cereals	16	Milk & milk products	16	Fats	16
Cereals	21	Beverages	14	Fats	16	Beverages	14	Milk & milk products	14
Fats	6	Fruits	14	Meat	11	Cereals	13	Meat	12
Beverages	5	Milk & Milk products	12	Milk & milk products	11	Fruits	11	Vegetables & vegetable products	12

Food groups and food items are listed in Supplementary online material—Table S1. Data of 2563 dietary records are used from 740 children at 3, 4, 5, 6, and 8 years of age and from 5 countries (Belgium, Germany, Italy, Poland, and Spain)

In Table 4, the frequency of skipping an EO is presented. Morning snack showed the highest frequency of skipping. Around 24% of dietary records reported no morning snack. All other EOs showed only a small number of skipping.

**Table 4:** Frequency of skipping an eating occasion on all record days based on total number of dietary protocols (*N*_protocol_ = 2563) of all comprised subjects (*N* = 740) and time points (3 to 8 years)

	*N*_protocol_	%
Breakfast	14	0.5
Morning snack	607	23.7
Lunch	3	0.1
Afternoon snack	40	1.6
Supper	3	0.1

**Fig. 3:**
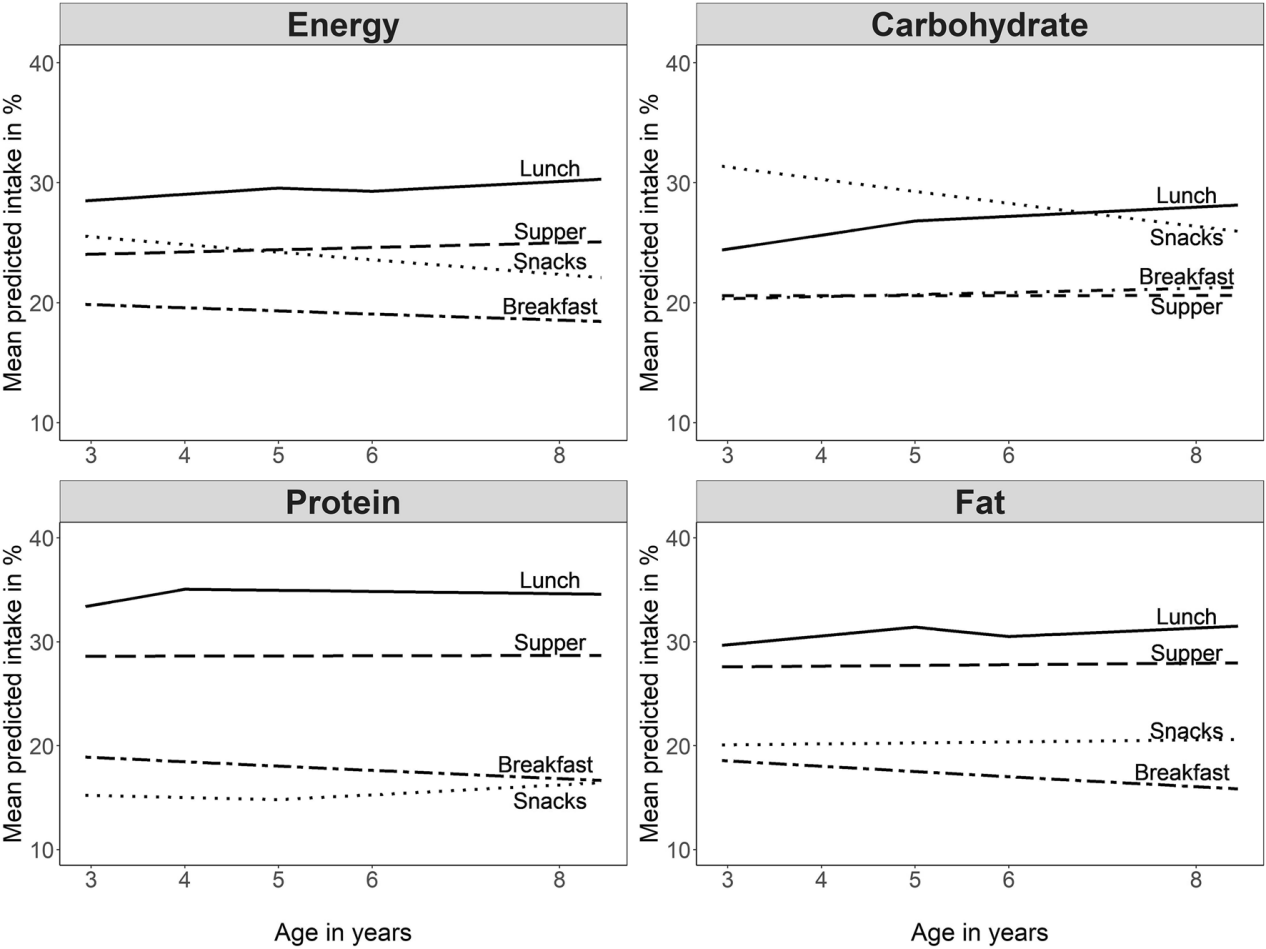
Mean predicted percentage energy and macronutrient energy percentages for eating occasions by age in children followed at 3, 4, 5, 6, and 8 years of age (*N* = 740). Data are based on beta regression (logit link) and applied to generalized linear mixed-effects models adjusted for misreporting, total energy intake (TEI), country and interaction of country, and TEI (for nutrients, TEI replaced by total nutrient intake)

Age trends are shown in Fig. 3. Predicted energy intake at snacks decreased significantly from 26 to 22% from 3 to 8 years (*p* < 0.001), along with a decreasing carbohydrate intake (*p* < 0.001). Predicted intake of energy from carbohydrate at lunch increased significantly over time from 24 to 28% (*p* < 0.001), whereas predicted intakes of energy from protein and fat at breakfast decreased from 19° to 17% and from 19 to 16%, respectively (*p* < 0.001). Numerical data to all figures are presented in the Supplementary Tables 4, 5, 6 and 7. Sensitivity analyses yielded similar results, except for energy intake at lunch and supper with significantly higher intakes at later ages (*p* < 0.0125; data not shown).

Boys and girls differed in energy and energy from macronutrient intakes, with boys over all ages consuming on average 73.6 kcal more than girls. However, no relative difference was noticed at each EO (all *p* > 0.05; data not shown).

## Discussion

The time-related distribution of energy and macronutrient intakes throughout the day of 740 healthy European children followed from 3 to 8 years of age showed highest mean energy intakes at lunch and supper. Breakfast and snacks had high proportions of carbohydrates, whereas lunch and supper were characterized by high-fat and protein intakes. Food intakes changed with age, with lower intakes for snacks and higher intakes for lunch and supper at later ages. No sex differences between EOs were observed.

The average energy intake consumed in this study population was in line with the recommended intakes (RI) for energy and nutrients defined by the European Food Safety Authority [28], except for total fat. For children aged 4 years and older, a total fat intake of 20–35%E is recommended [29, 30]. In the current cohort, total fat intake was at the upper limit or above the recommended range. Fat intakes above RI might contribute to a high-energy intake and to promotion of weight gain and an increased risk of overweight and obesity [29]. In this study population, energy intake from fat was highest during lunch and supper, where particular opportunities for reducing or changing fat intake may exist. Especially in Spain, fat %E was above the reference intake, and correspondently, carbohydrate %E was at the lower end of the recommended range, which agrees with result of another study examining Spanish children and adolescents [31]. The Spanish diet as part of the Mediterranean diet is usually characterized by high total fat intakes with high consumption of olive oil and a low proportion of saturated fats [32]. A Mediterranean diet supplemented with olive oil or nuts was found to be beneficial for health [33], and thus, the high fat intake Spanish children may not be an indicator for an unhealthy eating pattern. Since the fat profile was not examined, the present study does not support conclusions about quality of fat intake.

The timing, frequency and kind of food consumed depend on social and cultural aspects [9]. Furthermore, there is no unique definition of what is considered a meal or a snack [9, 34]. These aspects need to be taken into consideration when results of time-varying food intake are compared. A similar energy and nutrient (%E) distribution as in our study was also reported in other reports on Spanish children and adolescents [12] and Belgian adolescents [35]. However, Belgian children observed in our study consumed foods at lunch and supper of similar size. The different distribution could be due to the different age groups studied. In other countries not examined in our analysis, energy intake for lunch was reported lower than for supper in New Zealand [15, 36] and USA [37].

Snacking in younger children was prevalent in our study and contributed 24% of TEI. In other studies, snacking contributed from 20 to 24% [35] to 30.5% [38]. Vatanparast et al. [39] reported that younger children do not only snack more frequently but also consume more energy at snacks than adults. Snacking is an easy way for children to achieve energy balance. Young children have the highest energy and nutrient requirements based on their body weight than in any other phase of the lifespan [40]. Therefore, snacking may help young children to meet their relatively high-energy needs when gastric capacity is limited. The high carbohydrate portion at snacks (%E_Snacks_) related to frequent consumption of high-sugar-content foods observed in our study population seems to be concerning; however, total carbohydrate intake (%E) is within the recommended intake ranges of 45–60%E [30].

Although energy intake between girls and boys varied, we found no proportional differences either in daily energy intake or in the macronutrient distribution at each EO. Given that the energy difference between girls and boys was largest at 8 years of age, differences might evolve in older age groups. However, other studies also observed no sex differences in time-varying energy intake [15, 41].

Skipping of an EO is usually determined through questionnaires [2] or dietary records [14, 42]. Breakfast skipping in children was investigated in several previous studies, and a wide variation in prevalence was observed [43]. The variation in prevalence is also influenced by different definitions of breakfast skipping. When comparing our results with studies using a similar definition [3], breakfast skipping in children tends to be low. The percentage of persons skipping breakfast seems to increase in adolescence and adulthood [3, 14, 42].

Eating behaviour and macronutrient composition of EOs by age have been reported. A cross-sectional study performed in the UK showed similar energy and nutrient intakes during the life course (children, adolescents, adults and older adults) for breakfast [42]. Another cross-sectional study in New Zealand children found a larger proportion of daily energy intake during evening and a smaller proportion during afternoon in older children (11–14 years) compared to younger children (5–10 years) [15]. However, to our knowledge, age trends in a longitudinal setting and with the use of inferential statistics have not been examined. The decreased snack intake at later ages might be in line with decreased energy requirements based on body weight where less-frequent intakes are needed and more is eaten at main meals. Additionally, we found statistically significant results for breakfast (protein and fat); the effect sizes over time are small (e.g., mean predicted values vary from 3 to 8 years between 2 and 3 percentage points) indicating relatively stable eating habits over time.

### Strength and limitations

The strength of this study is its longitudinal design with a follow-up of children from 3 to 8 years of age. This design enabled us to study between and within subject variation. For data collection, 3-day weighed dietary protocols following standardized procedures were used, which allowed for a more accurate description of dietary intake than other methods. Furthermore, the present study comprised children from five European countries which increased the external validity of the study results and allowed studying cultural variation of eating habits. On the contrary, different cultural eating habits and time slots for typical EOs were challenging to standardize. All EOs were named the same for each country, although actual time points of the consumed meal might differ greatly. These standardisations were necessary for data analysis, but might hamper comparison between countries and eating pattern.

## Conclusion

The present study examined diurnal energy and energy from macronutrient intakes from early childhood to school age and revealed additional insights in the eating behaviour of children. Food was consumed regularly throughout the day and size of EOs seemed to be relatively stable at each age with highest intakes at lunch and supper. Possibly unhealthy EOs with high-fat intakes at lunch and supper and high-sugar-content foods at breakfast and snacks were observed, which may indicate potential for intervention. Practical approaches for a healthier diet may be achieved by substituting high-sugar-content foods with fruits, and by increasing the vegetable consumption at lunch and supper. Future studies should consider taking more detailed characterization of diet quality and critical nutrients across eating occasions into account.

## Supplementary Information

Below is the link to the electronic supplementary material.

Supplementary file1 (DOCX 15 KB)

Supplementary file2 (DOCX 14 KB)

Supplementary file3 (DOCX 16 KB)

Supplementary file4 (DOCX 22 KB)

Supplementary file5 (DOCX 26 KB)

Supplementary file6 (DOCX 24 KB)

Supplementary file7 (DOCX 27 KB)

## Abbreviations

BMI: Body Mass Index
CV: Coefficient of variation
EO: Eating occasion
TEI: Total energy intake
