# Supplementary material for: Distribution of energy and macronutrient intakes across eating occasions in European children from 3 to 8 years of age: The EU Childhood Obesity Project Study
Source: Eur J Nutr. 2022 Aug 5;62(1):165–74. doi: 10.1007/s00394-022-02944-6 (PMC9899743; doi:10.1007/s00394-022-02944-6)
Supplement: Supplementary file 1 — Supplementary file1 (DOCX 15 KB) [file 394_2022_2944_MOESM1_ESM.docx]

Table S1 Food groups and its main types of food components

| **FOOD GROUPS** | **FOOD GROUP CATEGORIES** |
| --- | --- |
| **CEREALS** | Whole grains |
|  | Refined grains |
|  | Processed cereal products |
| **EGGS** | Eggs |
| **FRUITS** | Malaceus and stone fruits |
|  | Berries & wild |
|  | Tropical fruits |
|  | Citrus |
|  | Processed fruit products |
| **VEGETABLES & VEGETABLE PRODUCTS** | Salad vegetables |
|  | Leafy green vegetables |
|  | Cruciferous vegetables |
|  | Others (sprout, root, others) |
|  | Fruiting vegetables |
|  | Leguminous vegetables |
|  | Soups and vegetable dishes |
| **LEGUMES** | Pulses |
| **NUTS & SEEDS** | Nuts & seeds |
| **INFANT FOODS** | Ready-to-eat infant foods |
| **SAVOURY SNACKS** | Savoury snacks |
| **POTATOES & STARCHY ROOTS** | Potatoes |
|  | Processed potatoes |
| **MILK & MILK PRODUCTS** | Cow's milk and regular yoghurt |
|  | Flavoured milk products |
|  | Processed milk products |
|  | Hard cheese |
|  | Soft cheese |
| **BEVERAGES** | Soft drinks |
|  | Caffeinated drinks |
|  | Alcohol drinks |
|  | Fruit juice |
|  | Teas |
| **WATER** | Water |
| **FATS** | Olive oil |
|  | Saturated spreads |
|  | Other oils and sauce |
| **SEASONINGS** | Non-caloric condiments |
|  | Other sauces (Not oil based) |
| **HIGH SUGAR CONTENT PRODUCTS** | Confectionery |
|  | Added sugars |
|  | Cakes, biscuits, sweet pastries |
| **FISH** | Lean fish and sea food |
|  | Fatty fish |
|  | Processed fish |
| **MEAT** | Red meat |
|  | White meat |
|  | Processed meat |
