# Supplementary material for: Distribution of energy and macronutrient intakes across eating occasions in European children from 3 to 8 years of age: The EU Childhood Obesity Project Study
Source: Eur J Nutr. 2022 Aug 5;62(1):165–74. doi: 10.1007/s00394-022-02944-6 (PMC9899743; doi:10.1007/s00394-022-02944-6)
Supplement: Supplementary file 2 — Supplementary file2 (DOCX 14 KB) [file 394_2022_2944_MOESM2_ESM.docx]

**Supplementary Table 2** Distribution of energy intake at eating occasions as a percentage of total intake in children followed at 3, 4, 5, 6 and 8 years of age; overall (N = 740) and by country (Belgium (N = 97), Germany (N = 106), Italy (N = 201), Poland (N = 126), Spain (N = 210)).

|  | **Total (kcal)** | **Breakfast** | **Morning snack** | **Lunch** | **Afternoon snack** | **Supper** | |
| --- | --- | --- | --- | --- | --- | --- | --- |
| Overall* | 1,340 ± 284 | 19 ± 7 | 8 ± 7 | 31 ± 8 | 16 ± 8 | 26 ± 8 | |
| Belgium | 1,253 ± 250 | 20 ± 7 | 10 ± 7 | 27 ± 7 | 17 ± 7 | 27 ± 7 | |
| Germany | 1,237 ± 297 | 22 ± 8 | 11 ± 7 | 26 ± 10 | 17 ± 9 | 24 ± 7 | |
| Italy | 1,305 ± 273 | 17 ± 6 | 3 ± 4 | 35 ± 8 | 14 ± 7 | 31 ± 7 | |
| Poland | 1,449 ± 239 | 22 ± 6 | 13 ± 9 | 30 ± 8 | 17 ± 9 | 19 ± 6 | |
| Spain | 1,403 ± 293 | 17 ± 6 | 8 ± 6 | 31 ± 7 | 19 ± 7 | 24 ± 7 | |
| Values are presented as mean ± standard deviation. If not otherwise indicated, values are presented as percentage of total energy intake. * Number of children is different for each country; overall average intake is not weighed for different number of subjects between countries. | | | | | | |  |
