# Supplementary material for: Distribution of energy and macronutrient intakes across eating occasions in European children from 3 to 8 years of age: The EU Childhood Obesity Project Study
Source: Eur J Nutr. 2022 Aug 5;62(1):165–74. doi: 10.1007/s00394-022-02944-6 (PMC9899743; doi:10.1007/s00394-022-02944-6)
Supplement: Supplementary file 3 — Supplementary file3 (DOCX 16 KB) [file 394_2022_2944_MOESM3_ESM.docx]

**Supplementary Table 3** Distribution of energy intake from macronutrients as a percentage of total energy intake at each eating occasion (%E_EO_) in children followed at 3, 4, 5, 6 and 8 years of age; overall (N = 740) and by country Belgium (N = 97), Germany (N = 106), Italy (N = 201), Poland (N = 126), Spain (N = 210)

|  | **Total**  **(kcal)** | **Breakfast**  **(%E_Breakfast_)** | **Lunch**  **(%E_Lunch_)** | **Supper**  **(%E_Supper_)** | **Snacks***  **(%E_Snacks_)** |
| --- | --- | --- | --- | --- | --- |
| **Overall**** |  |  |  |  |  |
| Carbohydrate | 681 ± 189 | 53 ± 12 | 45 ± 11 | 43 ± 13 | 61± 14 |
| Fat | 475 ± 136 | 32 ± 11 | 36 ± 9 | 39 ± 11 | 28 ± 11 |
| Protein | 203 ± 55 | 14 ± 4 | 18 ± 5 | 18 ± 6 | 9 ± 4 |
| **Belgium** |  |  |  |  |  |
| Carbohydrate | 687 ± 198 | 59 ± 12 | 49± 12 | 42 ± 12 | 63 ± 12 |
| Fat | 432 ± 118 | 27 ± 11 | 34 ± 11 | 39 ± 11 | 28 ± 11 |
| Protein | 181 ± 39 | 13 ± 4 | 17 ± 5 | 18 ± 5 | 8 ± 3 |
| **Germany** |  |  |  |  |  |
| Carbohydrate | 756 ± 352 | 57.± 11 | 51 ± 13 | 50 ± 13 | 64 ± 14 |
| Fat | 418 ± 123 | 29 ± 10 | 33 ± 11 | 36 ± 12 | 26 ± 11 |
| Protein | 169 ± 45 | 14 ± 4 | 15± 5 | 15 ± 5 | 9 ± 4 |
| **Italy** |  |  |  |  |  |
| Carbohydrate | 709 ± 179 | 53 ± 14 | 49 ± 9 | 47 ± 12 | 64 ± 16 |
| Fat | 446 ± 111 | 32 ± 11 | 34 ± 7 | 36 ± 10 | 25 ± 12 |
| Protein | 200 ± 45 | 14 ± 5 | 17 ± 4 | 17 ± 5 | 8 ± 3 |
| **Poland** |  |  |  |  |  |
| Carbohydrate | 736 ± 142 | 49 ± 11 | 42 ± 9 | 47 ± 11 | 63 ± 12 |
| Fat | 522 ± 105 | 37 ± 10 | 39 ± 7 | 38 ± 10 | 28 ± 10 |
| Protein | 207 ± 42 | 15 ± 3 | 19 ± 4 | 15 ± 3 | 9 ± 3 |
| **Spain** |  |  |  |  |  |
| Carbohydrate | 640 ± 146 | 52 ± 10 | 39 ± 9 | 34 ± 11 | 55 ± 10 |
| Fat | 559± 149 | 33 ± 9 | 40 ± 9 | 45 ± 10 | 33 ± 9 |
| Protein | 246 ± 59 | 14 ± 4 | 21 ± 5 | 21 ± 6 | 11 ± 3 |
| Values are presented as mean ± standard deviation. *Snack during morning and during afternoon are combined into “snacks” due to similar macronutrient distribution. ** “Number of children is different for each country; overall average intake is not weighed for different number of subjects between countries. | | | | | |
