# Supplementary material for: Distribution of energy and macronutrient intakes across eating occasions in European children from 3 to 8 years of age: The EU Childhood Obesity Project Study
Source: Eur J Nutr. 2022 Aug 5;62(1):165–74. doi: 10.1007/s00394-022-02944-6 (PMC9899743; doi:10.1007/s00394-022-02944-6)
Supplement: Supplementary file 4 — Supplementary file4 (DOCX 22 KB) [file 394_2022_2944_MOESM4_ESM.docx]

**Supplementary Table 4** Results of regression analysis (crude and adjusted models) of **energy intake** at eating occasions as a percentage of total energy intake by age in children followed at 3, 4, 5, 6 and 8 years of age (N = 732).

|  | **Breakfast** | | | | | **Lunch** | | | | | | | | | | | | **Supper** | | | | | **Snacks** | | | | | | | | | |
| --- | --- | --- | --- | --- | --- | --- | --- | --- | --- | --- | --- | --- | --- | --- | --- | --- | --- | --- | --- | --- | --- | --- | --- | --- | --- | --- | --- | --- | --- | --- | --- | --- |
| *Age* | | *Estimates* | *CI* | | *p* | | | |  | *Estimates* | | *CI* | *p* | | |  | *Estimates* | | *CI* | | | *p* | |  | *Estimates* | | | *CI* | | *p* | | |
| Crude model | | | | | | | | | | | | | | | | | | | | | | | | | | | | | | | | |
| Intercept | | -1.35 | -1.42 – -1.29 | **<0.001** | | |  | -0.89 | | | -0.93 – -0.86 | | **<0.001** |  | -1.16 | | | | | -1.22 – -1.11 | **<0.001** | | | | |  | -1.06 | | -1.13 – -0.99 | | **<0.001** |  |
| Age (in years)* | |  |  | |  | | | |  |  | |  |  | | |  |  | |  | | |  | |  |  | | |  | |  | | |
| 3-8 | | -0.03 | -0.04 – -0.01 | | **<0.001** | | | |  |  | |  |  | | |  | 0.02 | | 0.01 – 0.03 | | | **0.001** | |  | -0.03 | | | -0.04 – -0.01 | | **<0.001** | | |
| 3-5 | |  |  | |  | | | |  | 0.10 | | 0.06 – 0.15 | **<0.001** | | |  |  | |  | | |  | |  |  | | |  | |  | | |
| 5-6 | |  |  | |  | | | |  | 0.08 | | 0.04 – 0.12 | **<0.001** | | |  |  | |  | | |  | |  |  | | |  | |  | | |
| 6-8 | |  |  | |  | | | |  | 0.11 | | 0.06 – 0.17 | **<0.001** | | |  |  | |  | | |  | |  |  | | |  | |  | | |
| Adjusted model | | | | | | | | | | | | | | | | | | | | | | | | | | | | | | | | |
| Intercept | | -0.96 | -1.24 – -0.68 | | **<0.001** | | | |  | -1.18 | | -1.42 – -0.93 | **<0.001** | | |  | -1.15 | | -1.37 – -0.92 | | | **<0.001** | |  | -1.15 | | | -1.37 – -0.92 | | **<0.001** | | |
| Germany** | | -0.41 | -0.77 – -0.00 | | 0.025 | | | |  | -0.04 | | -0.36 – 0.28 | 0.796 | | |  | -0.04 | | -0.34 – 0.25 | | | 0.788 | |  | -0.04 | | | -0.34 – 0.25 | | 0.788 | | |
| Italy | | -0.40 | -0.73 – -0.08 | | 0.015 | | | |  | 0.47 | | 0.20 – 0.75 | **0.001** | | |  | 0.00 | | -0.00 – 0.00 | | | 0.814 | |  | -0.66 | | | -1.04 – -0.28 | | **0.001** | | |
| Poland | | -0.04 | -0.44 – 0.35 | | 0.827 | | | |  | 0.28 | | -0.07 – 0.62 | 0.118 | | |  | -0.35 | | -0.69 – -0.01 | | | **0.042** | |  | -0.08 | | | -0.54 – 0.38 | | 0.734 | | |
| Spain | | -0.26 | -0.59 – 0.07 | | 0.121 | | | |  | 0.37 | | 0.09 – 0.66 | **0.010** | | |  | -0.23 | | -0.49 – 0.04 | | | 0.090 | |  | -0.06 | | | -0.44 – 0.32 | | 0.748 | | |
| TEI*** | | -0.00 | -0.00 – -0.00 | | **0.012** | | | |  | 0.00 | | -0.00 – 0.00 | 0.342 | | |  | 0.00 | | -0.00 – 0.00 | | | 0.487 | |  | 0.00 | | | -0.00 – 0.00 | | 0.470 | | |
| TEI*Germany | | 0.00 | 0.00 – 0.00 | | **0.004** | | | |  | -0.00 | | -0.00 – 0.00 | 0.839 | | |  | -0.00 | | -0.00 – 0.00 | | | 0.490 | |  | 0.00 | | | -0.00 – 0.00 | | 0.814 | | |
| TEI*Italy | | 0.00 | -0.00 – 0.00 | | 0.268 | | | |  | -0.00 | | -0.00 – 0.00 | 0.573 | | |  | 0.00 | | -0.00 – 0.00 | | | 0.246 | |  | 0.00 | | | -0.00 – 0.00 | | 0.837 | | |
| TEI*Poland | | 0.00 | -0.00 – 0.00 | | 0.332 | | | |  | -0.00 | | -0.00 – 0.00 | 0.442 | | |  | -0.00 | | -0.00 – 0.00 | | | 0.444 | |  | 0.00 | | | -0.00 – 0.00 | | 0.273 | | |
| TEI*Spain | | 0.00 | -0.00 – 0.00 | | 0.598 | | | |  | -0.00 | | -0.00 – 0.00 | 0.257 | | |  | 0.00 | | -0.00 – 0.00 | | | 0.464 | |  | 0.00 | | | -0.00 – 0.00 | | 0.522 | | |
| Underreport**** | | 0.02 | -0.04 – 0.08 | | 0.488 | | | |  | 0.03 | | -0.03 – 0.08 | 0.318 | | |  | -0.00 | | -0.05 – 0.05 | | | 0.957 | |  | -0.11 | | | -0.19 – -0.03 | | **0.005** | | |
| Overreport | | 0.02 | -0.05 – 0.09 | | 0.554 | | | |  | -0.03 | | -0.09 – 0.02 | 0.245 | | |  | -0.04 | | -0.10 – 0.01 | | | 0.135 | |  | 0.05 | | | -0.02 – 0.13 | | 0.183 | | |
| Age (in years)* | |  |  | |  | | | |  |  | |  |  | | |  |  | |  | | |  | |  |  | | |  | |  | | |
| 3-8 | | -0.02 | -0.03 – -0.00 | | 0.033 | | | |  |  | |  |  | | |  | 0.01 | | -0.00 – 0.02 | | | 0.106 | |  | -0.03 | | | -0.05 – -0.02 | | **<0.001** | | |
| 3-5 | |  |  | |  | | | |  | 0.05 | | 0.00 – 0.10 | **0.041** | | |  |  | |  | | |  | |  |  | | |  | |  | | |
| 5-6 | |  |  | |  | | | |  | 0.04 | | -0.01 – 0.09 | 0.148 | | |  |  | |  | | |  | |  |  | | |  | |  | | |
| 6-8 | |  |  | |  | | | |  | 0.09 | | 0.01 – 0.16 | **0.022** | | |  |  | |  | | |  | |  |  | | |  | |  | | |
| Results of beta regression (logit link) applied to generalized linear mixed effects models with random intercept per subject and random slope varying with age. P = 0.0125 (equivalent to P<0.05 after Bonferroni correction).*Piecewise linear splines of age instead of linear age were added for lunch with knots at 5 and 6 years ** All effects for countries in reference to Belgium; ***TEI = Total energy intake (kcal); ****All effects for misreport in reference to plausible report of total energy intake. | | | | | | | | | | | | | | | | | | | | | | | | | | | | | | | | |
