# Supplementary material for: Distribution of energy and macronutrient intakes across eating occasions in European children from 3 to 8 years of age: The EU Childhood Obesity Project Study
Source: Eur J Nutr. 2022 Aug 5;62(1):165–74. doi: 10.1007/s00394-022-02944-6 (PMC9899743; doi:10.1007/s00394-022-02944-6)
Supplement: Supplementary file 5 — Supplementary file5 (DOCX 26 KB) [file 394_2022_2944_MOESM5_ESM.docx]

**Supplementary Table 5** Results of regression analysis (crude and adjusted models) of energy intake from **carbohydrate** at eating occasions as a percentage of total energy intake (%E) by age in children followed at 3, 4, 5, 6 and 8 years of age (N = 732)

|  | **Breakfast** | | | | **Lunch** | | | | | | | **Supper** | | | | | **Snacks** | | |  |  |  |
| --- | --- | --- | --- | --- | --- | --- | --- | --- | --- | --- | --- | --- | --- | --- | --- | --- | --- | --- | --- | --- | --- | --- |
| *Age* | | *Estimates* | *CI* | *p* | |  | *Estimates* | *CI* | *p* |  | *Estimates* | | *CI* | *p* |  | *Estimates* | | *CI* | *p* | |  |  |
| Crude model | | | | | | | | | | | | | | | | | | | | | | |
| Intercept | | -1.38 | -1.45 – -1.31 | **<0.001** | |  | -1.05 | -1.09 – -1.01 | **<0.001** |  | -1.38 | | -1.45 – -1.32 | **<0.001** |  | -0.70 | | -0.77 – -0.62 | **<0.001** | | |  |
| Age (in years)* | |  |  |  | |  |  |  |  |  |  | |  |  |  |  | |  |  | | |  |
| 3-8 | | -0.00 | -0.02 – 0.01 | 0.548 | |  |  |  |  |  | 0.02 | | 0.00 – 0.03 | **0.008** |  | -0.04 | | -0.06 – -0.03 | **<0.001** | | |  |
| 3-5 | |  |  |  | |  | 0.11 | 0.06 – 0.16 | **<0.001** |  |  | |  |  |  |  | |  |  | | |  |
| 5-8 | |  |  |  | |  | 0.14 | 0.07 – 0.20 | **<0.001** |  |  | |  |  |  |  | |  |  | | |  |
| Adjusted model | | | | | | | | | | | | | | | | | | | | | | |
| Intercept | | -0.86 | -1.11 – -0.62 | **<0.001** | |  | -1.29 | -1.51 – -1.07 | **<0.001** |  | -1.45 | | -1.67 – -1.24 | **<0.001** |  | -0.71 | | -0.96 – -0.45 | **<0.001** | | |  |
| Germany** | | -0.48 | -0.77 – -0.18 | **0.001** | |  | 0.04 | -0.22 – 0.30 | 0.747 |  | 0.15 | | -0.12 – 0.41 | 0.278 |  | 0.06 | | -0.26 – 0.38 | 0.712 | | |  |
| Italy | | -0.62 | -0.91 – -0.34 | **<0.001** | |  | 0.70 | 0.46 – 0.95 | **<0.001** |  | 0.12 | | -0.12 – 0.37 | 0.331 |  | -0.66 | | -0.96 – -0.35 | **<0.001** | | |  |
| Poland | | -0.28 | -0.65 – 0.09 | 0.136 | |  | 0.30 | -0.02 – 0.63 | 0.067 |  | 0.05 | | -0.30 – 0.39 | 0.800 |  | -0.11 | | -0.49 – 0.28 | 0.586 | | |  |
| Spain | | -0.33 | -0.63 – -0.03 | **0.031** | |  | 0.30 | 0.04 – 0.57 | **0.022** |  | -0.28 | | -0.55 – -0.00 | **0.047** |  | 0.03 | | -0.29 – 0.34 | 0.866 | | |  |
| CHO*** | | -0.00 | -0.00 – -0.00 | **0.001** | |  | 0.00 | -0.00 – 0.00 | 0.645 |  | 0.00 | | -0.00 – 0.00 | 0.350 |  | 0.00 | | -0.00 – 0.00 | 0.227 | | |  |
| CHO*Germany | | 0.00 | 0.00 – 0.00 | **0.001** | |  | -0.00 | -0.00 – 0.00 | 0.378 |  | -0.00 | | -0.00 – 0.00 | 0.292 |  | -0.00 | | -0.00 – 0.00 | 0.809 | | |  |
| CHO *Italy | | 0.00 | -0.00 – 0.00 | 0.053 | |  | -0.05 | -0.00 – -0.00 | **0.039** |  | 0.00 | | -0.00 – 0.00 | 0.069 |  | 0.00 | | -0.00 – 0.00 | 0.818 | | |  |
| CHO *Poland | | 0.00 | -0.00 – 0.00 | 0.249 | |  | -0.00 | -0.00 – 0.00 | 0.099 |  | -0.00 | | -0.00 – 0.00 | 0.082 |  | 0.00 | | -0.00 – 0.00 | 0.071 | | |  |
| CHO *Spain | | 0.00 | -0.00 – 0.00 | 0.311 | |  | -0.00 | -0.00 – 0.00 | 0.146 |  | 0.00 | | -0.00 – 0.00 | 0.420 |  | 0.00 | | -0.00 – 0.00 | 0.388 | | |  |
| Underreport**** | | 0.05 | -0.02 – 0.11 | 0.173 | |  | 0.02 | -0.04 – 0.08 | 0.586 |  | -0.05 | | -0.12 – 0.01 | 0.110 |  | -0.14 | | -0.21 – -0.06 | **<0.001** | | |  |
| Overreport | | 0.03 | -0.04 – 0.10 | 0.394 | |  | -0.02 | -0.08 – 0.04 | 0.546 |  | -0.06 | | -0.12 – 0.01 | 0.102 |  | 0.01 | | -0.07 – 0.08 | 0.811 | | |  |
| Age (in years)* | |  |  |  | |  |  |  |  |  |  | |  |  |  |  | |  |  | | |  |
| 3-8 | | 0.00 | -0.02 – 0.02 | 0.978 | |  |  |  |  |  | 0.01 | | -0.00 – 0.02 | 0.139 |  | -0.05 | | -0.06 – -0.03 | **<0.001** | | |  |
| 3-5 | |  |  |  | |  | 0.13 | 0.07 – 0.18 | **<0.001** |  |  | |  |  |  |  | |  |  | | |  |
| 5-8 | |  |  |  | |  | 0.19 | 0.11 – 0.27 | **<0.001** |  |  | |  |  |  |  | |  |  | | |  |
| Results of beta regression (logit link) applied to generalized linear mixed effects models with random intercept per subject and random slope varying with age. P = 0.0125 (equivalent to P<0.05 after Bonferroni correction). * Piecewise linear splines of age instead of linear age were added for lunch with knots at 5 and 6 years** All effects for countries in reference to Belgium *** CHO = Total carbohydrate intake (kcal); ****All effects for misreport in reference to plausible report of total energy intake. | | | | | | | | | | | | | | | | | | | | | | |
