# Supplementary material for: Distribution of energy and macronutrient intakes across eating occasions in European children from 3 to 8 years of age: The EU Childhood Obesity Project Study
Source: Eur J Nutr. 2022 Aug 5;62(1):165–74. doi: 10.1007/s00394-022-02944-6 (PMC9899743; doi:10.1007/s00394-022-02944-6)
Supplement: Supplementary file 6 — Supplementary file6 (DOCX 24 KB) [file 394_2022_2944_MOESM6_ESM.docx]

**Supplementary Table 6** Results of regression analysis (crude and adjusted models) of energy intake from **protein** at eating occasions as a percentage of total energy intake (%E) by age in children followed at 3, 4, 5, 6 and 8 years of age (N = 732).

| **Breakfast** | | | | | | | | | | | **Lunch** | | | **Supper** | | | | | | | | | | | | | | | | | | | **Snacks** | | | | |  |  |
| --- | --- | --- | --- | --- | --- | --- | --- | --- | --- | --- | --- | --- | --- | --- | --- | --- | --- | --- | --- | --- | --- | --- | --- | --- | --- | --- | --- | --- | --- | --- | --- | --- | --- | --- | --- | --- | --- | --- | --- |
| *Age* | *Estimates* | *CI* | *p* | |  | | *Estimates* | | | *CI* | | | *p* | | |  | | *Estimates* | | | *CI* | | *p* | | |  | | | | *Estimates* | | | *CI* | *p* | | | |  |  |
| Crude model | | | | | | | | | | | | | | | | | | | | | | | | | | | | | | | | | | | | | |  |  |
| Intercept | -1.31 | -1.38 – -1.24 | **<0.001** | |  | | -0.66 | | -0.71 – -0.62 | | | **<0.001** | | |  | | -0.97 | | -1.03 – -0.91 | | | | | **<0.001** | |  | | | -1.70 | | | -1.75 – -1.64 | | | | | **<0.001** |  |  |
| Age (in years)* |  |  |  | |  | |  | |  | | |  | | |  | |  | |  | | | | |  | |  | | |  | | |  | | | | |  |  |  |
| 3-8 | -0.05 | -0.07 – -0.04 | **<0.001** | |  | |  | |  | | |  | | |  | | 0.02 | | 0.01 – 0.03 | | | | | **0.001** | |  | | |  | | |  | | | | |  |  |  |
| 3-5 |  |  |  | |  | |  | |  | | |  | | |  | |  | |  | | | | |  | |  | | | -0.09 | | | -0.15 – -0.03 | | | | | **0.005** |  |  |
| 5-8 |  |  |  | |  | |  | |  | | |  | | |  | |  | |  | | | | |  | |  | | | -0.05 | | | -0.13 – 0.02 | | | | | 0.179 |  |  |
| 3-4 |  |  |  | |  | | 0.10 | | 0.05 – -0.14 | | | **<0.001** | | |  | |  | |  | | | | |  | |  | | |  | | |  | | | | |  |  |  |
| 4-8 |  |  |  | |  | | 0.13 | | 0.07 – 0.19 | | | **<0.001** | | |  | |  | |  | | | | |  | |  | | |  | | |  | | | | |  |  |  |
| Adjusted model | | | | | | | | | | | | | | | | | | | | | | | | | | | | | | | | | | | | | |  |  |
| Intercept | **<0.001** | **<0.001** | | **<0.001** | |  | | -1.04 | | -1.29 – -0.79 | | | **<0.001** | | |  | | -0.73 | | -0.97 – -0.49 | | **<0.001** | | | | |  | -0.73 | | | | -0.97 – -0.49 | | | | **<0.001** | |  |  |
| Germany** | 0.27 | -0.08 – 0.62 | | 0.132 | |  | | -0.11 | | -0.43 – 0.21 | | | 0.505 | | |  | | -0.42 | | -0.73 – -0.11 | | **0.008** | | | | |  | -0.10 | | | | -0.53 – 0.33 | | | | 0.648 | |  |  |
| Italy | 0.14 | -0.19 – 0.46 | | 0.408 | |  | | 0.55 | | 0.26 – 0.84 | | | **<0.001** | | |  | | -0.41 | | -0.69 – -0.13 | | **0.004** | | | | |  | -0.62 | | | | -1.03 – -0.20 | | | | **0.004** | |  |  |
| Poland | 0.24 | -0.12 – 0.60 | | 0.192 | |  | | 0.32 | | -0.01 – 0.65 | | | 0.054 | | |  | | -0.78 | | -1.12 – -0.45 | | **<0.001** | | | | |  | -0.00 | | | | -0.44 – 0.44 | | | | 0.996 | |  |  |
| Spain | 0.22 | -0.11 – 0.54 | | 0.188 | |  | | 0.23 | | -0.06 – 0.52 | | | 0.113 | | |  | | -0.55 | | -0.83 – -0.27 | | **<0.001** | | | | |  | 0.12 | | | | -0.27 – 0.51 | | | | 0.554 | |  |  |
| Protein*** | -0.00 | -0.00 – -0.00 | | 0.336 | |  | | 0.00 | | -0.00 – 0.00 | | | 0.137 | | |  | | 0.00 | | -0.00 – 0.00 | | 0.537 | | | | |  | -0.00 | | | | -0.00 – 0.00 | | | | 0.015 | |  |  |
| Protein*Germany | -0.00 | -0.00 – 0.00 | | 0.688 | |  | | 0.00 | | -0.00 – 0.00 | | | 0.933 | | |  | | 0.00 | | -0.00 – 0.00 | | 0.682 | | | | |  | 0.00 | | | | -0.00 – 0.00 | | | | 0.052 | |  |  |
| Protein*Italy | -0.00 | -0.00 – -0.00 | | 0.016 | |  | | -0.00 | | -0.00 – 0.00 | | | 0.231 | | |  | | 0.00 | | 0.00 – 0.00 | | **0.002** | | | | |  | 0.00 | | | | -0.00 – 0.00 | | | | 0.639 | |  |  |
| Protein*Poland | 0.00 | -0.00 – 0.00 | | 0.908 | |  | | -0.00 | | -0.00 – 0.00 | | | 0.939 | | |  | | 0.00 | | -0.00 – 0.00 | | 0.991 | | |  | 0.00 | | | | | -0.00 – 0.00 | | | | 0.159 | | | |  |
| Protein*Spain | -0.00 | -0.00 – -0.00 | | 0.015 | |  | | 0.00 | | -0.00 – 0.00 | | | 0.964 | | |  | | 0.00 | | -0.00 – 0.00 | | 0.098 | | | | |  | 0.00 | | | | -0.00 – 0.00 | | | | 0.280 | |  |  |
| Underreport**** | -0.05 | -0.11 – 0.01 | | 0.100 | |  | | 0.02 | | -0.04 – 0.07 | | | 0.497 | | |  | | 0.07 | | 0.02 – 0.13 | | **0.008** | | | | |  | -0.23 | | | | -0.31 – -0.15 | | | | **<0.001** | |  |  |
| Overreport | 0.05 | -0.02 – 0.11 | | 0.137 | |  | | -0.09 | | -0.14 – -0.03 | | | **0.003** | | |  | | -0.05 | | -0.11 – 0.01 | | 0.088 | | | | |  | 0.17 | | | | 0.09 – 0.25 | | | | **<0.001** | |  |  |
| Age (in years)* |  |  | |  | |  | |  | |  | | |  | | |  | |  | |  | |  | | | | |  |  | | | |  | | | |  | |  |  |
| 3-8 | -0.03 | -0.04 – -0.01 | | **<0.001** | |  | |  | |  | | |  | | |  | | 0.00 | | -0.01 – 0.01 | | 0.922 | | | | |  |  | | | |  | | | |  | |  |  |
| 3-5 |  |  | |  | |  | |  | |  | | |  | | |  | |  | |  | |  | | | | |  | -0.03 | | | | -0.10 – 0.03 | | | | 0.314 | |  |  |
| 5-8 |  |  | |  | |  | |  | |  | | |  | | |  | |  | |  | |  | | | | |  | 0.09 | | | | 0.00 – 0.18 | | | | **0.050** | |  |  |
| 3-4 |  |  | |  | |  | | 0.07 | | 0.03 – 0.12 | | | **0.002** | | |  | |  | |  | |  | | | | |  |  | | | |  | | | |  | |  |  |
| 4-8 |  |  | |  | |  | | 0.05 | | -0.02 – 0.12 | | | 0.148 | | |  | |  | |  | |  | | | | |  |  | | | |  | | | |  | |  |  |
| Results of beta regression (logit link) applied to generalized linear mixed effects models with random intercept per subject and random slope varying with age. P = 0.0125 (equivalent to P<0.05 after Bonferroni correction).* Piecewise linear splines were added for snacks with knot at 5 years** All effects for countries in reference to Belgium *** Protein = Total protein intake (kcal); ****All effects for misreport in reference to plausible report of total energy intake. | | | | | | | | | | | | | | | | | | | | | | | | | | | | | | | | | | | | | | | |
