# Supplementary material for: Distribution of energy and macronutrient intakes across eating occasions in European children from 3 to 8 years of age: The EU Childhood Obesity Project Study
Source: Eur J Nutr. 2022 Aug 5;62(1):165–74. doi: 10.1007/s00394-022-02944-6 (PMC9899743; doi:10.1007/s00394-022-02944-6)
Supplement: Supplementary file 7 — Supplementary file7 (DOCX 27 KB) [file 394_2022_2944_MOESM7_ESM.docx]

**Supplementary Table 7** Results of regression analysis (crude and adjusted models) of energy intake form **fat** at eating occasions as a percentage of total energy intake (%E) by age in children followed at 3, 4, 5, 6 and 8 years of age (N = 732).

|  | **Breakfast** | | | | | | | | | | | | | | **Lunch** | | | | | | | **Supper** | | | | | | | | | | | | | | | | | | | **Snacks** | | | | | | | | | |
| --- | --- | --- | --- | --- | --- | --- | --- | --- | --- | --- | --- | --- | --- | --- | --- | --- | --- | --- | --- | --- | --- | --- | --- | --- | --- | --- | --- | --- | --- | --- | --- | --- | --- | --- | --- | --- | --- | --- | --- | --- | --- | --- | --- | --- | --- | --- | --- | --- | --- | --- |
| *Age* | | *Estimates* | | | | | *CI* | | *p* |  | | | | *Estimates* | | | | *CI* | | | *p* | | | |  | | *Estimates* | | | *CI* | *p* | | | | |  | | | | *Estimates* | | | | *CI* | *p* | | | | |  |
| Crude model | | | | | | | | | | | | | | | | | | | | | | | | | | | | | | | | | | | | | | | | | | | | | | | | | | |
| Intercept | | | -1.34 | | | -1.43 – -1.26 | | **<0.001** | | |  | -0.82 | | | | -0.86 – -0.77 | | | | **<0.001** | | | |  | | -0.99 | | | -1.06 – -0.93 | | | **<0.001** | | | |  | | | -1.44 | | | | -1.54– -1.35 | | | **<0.001** | |  |  |  |
| Age (in years)* | | | |  |  | | |  | |  | | |  | | | |  | |  | | | |  |  | | | |  | | | | |  | |  | | | |  | | | |  | | |  | |  |  |  |
| 3-8 | | | -0.05 | | | -0.06 – -0.03 | | **<0.001** | | |  |  | | | |  | | | |  | | | |  | | 0.01 | | | 0.00 – 0.03 | | | **0.020** | | | |  | | | 0.00 | | | | -0.01 – 0.02 | | | 0.545 | |  |  |  |
| 3-5 | | |  | | |  | |  | | |  | 0.10 | | | | 0.04 – 0.16 | | | | **0.001** | | | |  | |  | | |  | | |  | | | |  | | |  | | | |  | | |  | |  |  |  |
| 5-6 | | |  | | |  | |  | | |  | 0.05 | | | | -0.01 – 0.11 | | | | 0.084 | | | |  | |  | | |  | | |  | | | |  | | |  | | | |  | | |  | |  |  |  |
| 6-8 | | |  | | |  | |  | | |  | 0.09 | | | | 0.01 – 0.17 | | | | **0.020** | | | |  | |  | | |  | | |  | | | |  | | |  | | | |  | | |  | |  |  |  |
| Adjusted model | | | | | | | | | | | | | | | | | | | | | | | | | | | | | | | | | | | | | | | | | | | | | | | | | | |
| Intercept | | | -1.26 | | | -1.54 – -0.99 | | **<0.001** | | |  | -1.20 | | | | -1.43 – -0.97 | | | | **<0.001** | | | |  | | -0.84 | | | -1.06 – -0.63 | | | **<0.001** | | | |  | | -1.32 | | | | | -1.63 – -1.02 | | | | **<0.001** | | | |
| Germany** | | | 0.09 | | | -0.28 – 0.46 | | 0.635 | | |  | 0.24 | | | | -0.07 – 0.55 | | | | 0.127 | | | |  | | -0.38 | | | -0.68 – -0.08 | | | **0.014** | | | |  | | -0.22 | | | | | -0.64 – 0.20 | | | | 0.299 | | | |
| Italy | | | -0.06 | | | -0.39 – 0.26 | | 0.706 | | |  | 0.65 | | | | 0.38 – 0.92 | | | | **<0.001** | | | |  | | -0.17 | | | -0.42 – 0.09 | | | 0.199 | | | |  | | -0.67 | | | | | -1.05 – -0.30 | | | | **<0.001** | | | |
| Poland | | | 0.30 | | | -0.11 – 0.70 | | 0.155 | | |  | 0.50 | | | | 0.15 – 0.85 | | | | **0.005** | | | |  | | -0.62 | | | -0.98 – -0.27 | | | **0.001** | | | |  | | -0.24 | | | | | -0.70 – 0.22 | | | | 0.307 | | | |
| Spain | | | -0.05 | | | -0.39 – 0.28 | | 0.754 | | |  | 0.43 | | | | 0.16 – 0.70 | | | | **0.002** | | | |  | | -0.34 | | | -0.60 – -0.08 | | | **0.010** | | | |  | | 0.16 | | | | | -0.21 – 0.52 | | | | 0.399 | | | |
| Fat*** | | | -0.00 | | | -0.00 – 0.00 | | 0.121 | | |  | 0.00 | | | | -0.00 – 0.00 | | | | 0.119 | | | |  | | 0.00 | | | -0.00 – 0.00 | | | 0.588 | | | |  | | 0.00 | | | | | -0.00 – 0.00 | | | | 0.890 | | | |
| Fat*Germany | | | 0.00 | | | -0.00 – 0.00 | | 0.393 | | |  | -0.00 | | | | -0.00 – -0.00 | | | | 0.056 | | | |  | | 0.00 | | | -0.00 – 0.00 | | | 0.389 | | | |  | | 0.00 | | | | | -0.00 – 0.00 | | | | 0.172 | | | |
| Fat*Italy | | | 0.00 | | | -0.00 – 0.00 | | 0.700 | | |  | -0.00 | | | | -0.00 – 0.00 | | | | 0.069 | | | |  | | 0.00 | | | -0.00 – 0.00 | | | 0.052 | | | |  | | 0.00 | | | | | -0.00 – 0.00 | | | | 0.967 | | | |
| Fat*Poland | | | 0.00 | | | -0.00 – 0.00 | | 0.478 | | |  | -0.00 | | | | -0.00 – 0.00 | | | | 0.148 | | | |  | | 0.00 | | | -0.00 – 0.00 | | | 0.953 | | | |  | | 0.00 | | | | | -0.00 – 0.00 | | | | 0.209 | | | |
| Fat*Spain | | | 0.00 | | | -0.00 – 0.00 | | 0.943 | | |  | -0.00 | | | | -0.00 – 0.00 | | | | 0.104 | | | |  | | 0.00 | | | -0.00 – 0.00 | | | 0.258 | | | |  | | -0.00 | | | | | -0.00 – 0.00 | | | | 0.818 | | | |
| Underreport**** | | | -0.04 | | | -0.11 – 0.04 | | 0.368 | | |  | 0.03 | | | | -0.04 – 0.09 | | | | 0.391 | | | |  | | 0.04 | | | -0.02 – 0.11 | | | 0.208 | | | |  | | -0.19 | | | | | -0.28 – -0.09 | | | | **<0.001** | | | |
| Overreport | | | -0.00 | | | -0.08 – 0.08 | | 0.977 | | |  | -0.06 | | | | -0.13 – 0.01 | | | | 0.103 | | | |  | | -0.06 | | | -0.13 – 0.01 | | | 0.099 | | | |  | | 0.14 | | | | | 0.04 – 0.23 | | | | **0.005** | | | |
| Age (in years)* | | | |  |  | | |  | |  | | |  | | | |  | |  | | | |  |  | | | |  | | | | |  |  | | |  | | | | |  | | | | |  | |  |  |
| 3-8 | | | -0.03 | | | -0.05 – -0.02 | | **<0.001** | | |  |  | | | |  | | | |  | | | |  | | 0.01 | | | -0.00 – 0.02 | | | 0.655 | | | |  | | 0.01 | | | | | -0.01 – 0.03 | | | | 0.569 | | | |
| 3-5 | | |  | | |  | |  | | |  | 0.08 | | | | 0.02 – 0.15 | | | | **0.010** | | | |  | |  | | |  | | |  | | | |  | |  | | | | |  | | | |  | | | |
| 5-6 | | |  | | |  | |  | | |  | 0.04 | | | | -0.02 – 0.10 | | | | 0.221 | | | |  | |  | | |  | | |  | | | |  | |  | | | | |  | | | |  | | | |
| 6-8 | | |  | | |  | |  | | |  | 0.09 | | | | -0.00 – 0.18 | | | | 0.058 | | | |  | |  | | |  | | |  | | | |  | |  | | | | |  | | | |  | | | |
| Results of beta regression (logit link) applied to generalized linear mixed effects models with random intercept per subject and random slope varying with age. P = 0.0125 (equivalent to P<0.05 after Bonferroni correction).* Piecewise linear splines of age instead of linear age were added for lunch with knots at 5 and 6 years** All effects for countries in reference to Belgium *** Fat = Total fat intake (kcal); ****All effects for misreport in reference to plausible report of total energy intake. | | | | | | | | | | | | | | | | | | | | | | | | | | | | | | | | | | | | | | | | | | | | | | | | | | |
